# Radiomics-based interpretable machine learning model for identifying high-risk patients with small bowel obstruction: a multicenter study

**DOI:** 10.3389/fmed.2026.1848334

**Published:** 2026-10-09

**Authors:** Guozheng Zhang, Xiaohui Liu, Weitao Huang, Xisong Zhu, Cong Xia, Hua Xia, Yuguo Wei, Xiaowei Han, Danqiong Wang

**Affiliations:** 1 Department of Radiology, The Quzhou Affiliated Hospital of Wenzhou Medical University Quzhou People’s Hospital, Quzhou, China; 2 Department of Radiology, Quzhou Kecheng People’s Hospital, Quzhou, China; 3 Department of Radiology, Quzhou TCM Hospital at the Junction of Four Provinces, Quzhou, China; 4 Department of Medical Affairs, Pharmaceutical Diagnosis, GE Healthcare, Hangzhou, China; 5 Department of Critical Care Medicine, The Quzhou Affiliated Hospital of Wenzhou Medical University Quzhou People’s Hospital, Quzhou, China

**Keywords:** computed tomography, machine learning, radiomics, risk stratification, small bowel obstruction

## Abstract

**Background:**

Early identification of high-risk small bowel obstruction remains clinically challenging, especially when prompt intervention is needed to prevent bowel ischemia and related adverse outcomes.

**Methods:**

In this multicenter retrospective study, 274 patients with small bowel obstruction from three hospitals were included. Patients from Hospital A were randomly divided into training and test cohorts, and patients from the other two hospitals formed an external validation cohort. Radiomics features were extracted from venous-phase contrast-enhanced CT images, and logistic regression, naive Bayes, Extra Trees, Light Gradient Boosting Machine (LightGBM), and multilayer perceptron models were developed. Independent clinical predictors were identified and integrated with radiomics features to build a combined model.

**Results:**

LightGBM showed the most stable internal performance, with AUC values of 0.934 and 0.870 in the training and test cohorts, respectively. Lower diastolic blood pressure and bowel wall thickening were independent predictors of high-risk status. The combined model achieved AUC values of 0.942, 0.867, and 0.803 in the training, test, and external validation cohorts, respectively, with an external validation accuracy of 0.833 and sensitivity of 0.946.

**Conclusion:**

CT-based radiomics can identify patients with small bowel obstruction at high risk, and a combined model integrating radiomics and clinical predictors may support earlier risk stratification and intervention.

## Introduction

Small bowel obstruction (SBO) is a common acute abdominal emergency characterized by substantial clinical heterogeneity, and adhesive SBO accounts for a large proportion of cases (1–3). Although many patients improve with nonoperative management such as gastrointestinal decompression, fasting, and fluid resuscitation, some progress to bowel ischemia, necrosis, strangulation, or failure of conservative treatment and ultimately require emergency surgery (4–6). Delayed recognition of these high-risk patients increases complications, bowel loss, and mortality (7). In routine practice, this decision is often difficult because symptoms, laboratory results, and early imaging findings do not always evolve in parallel. Clinicians therefore need not only confirmation of obstruction, but also early tools that can separate patients likely to respond to conservative management from those who may deteriorate and require urgent escalation of care.

Computed tomography (CT) remains the cornerstone of imaging evaluation for SBO because it can identify the level, cause, and severity of obstruction while also depicting high-risk findings such as bowel ischemia, mesenteric involvement, and ascites (8–10). Previous studies have shown that bowel wall thickening, decreased enhancement, mesenteric edema, mesenteric fluid, and ascites are associated with strangulation or surgically relevant ischemia (9–14). However, qualitative CT interpretation still depends heavily on reader experience and may be challenged by subtle findings, overlapping inflammatory changes, and interobserver variability. In borderline cases, the visible CT signs may be insufficiently specific or may appear only after clinically important bowel compromise has already developed. As a result, conventional CT assessment alone may not fully capture the underlying biologic heterogeneity of obstructed bowel segments or provide sufficiently reproducible risk stratification across centers.

Radiomics extracts high-throughput quantitative features from medical images and provides a complementary analytic dimension for disease-risk assessment and treatment decision-making (15–17). Prior studies have demonstrated broad predictive potential for radiomics across multiple diseases, and increasing attention has been directed toward interpretability, generalizability, and clinical translatability (18–21). In SBO, previous investigations have attempted to predict failure of conservative management, strangulation, ischemia, and early surgical need using CT findings, clinical scores, inflammatory biomarkers, and early machine-learning approaches (22–25). Nevertheless, multicenter, interpretable, and externally validated radiomics models for identifying patients with SBO at high risk remain relatively scarce. This gap is clinically important because a model that performs only in a single-center development cohort, or that cannot explain why it flags a patient as high risk, is unlikely to influence real emergency decision-making.

Accordingly, we developed radiomics, clinical, and combined models based on CT radiomics features, conventional CT findings, and clinical variables and validated these models in a multicenter cohort. SHAP was further used to improve model interpretability and to provide quantitative evidence for the early identification of high-risk SBO. We specifically aimed to determine whether integrating radiomics with key clinical predictors could improve risk stratification beyond either approach alone and whether such performance could be retained in an external validation cohort drawn from other institutions. The distinguishing contribution of the present work is therefore not radiomics or machine learning alone, but their combination in a three-hospital design that directly compares five algorithms, evaluates clinical, radiomics, and combined models, uses SHAP for both global and patient-level explanations, and tests generalizability in an independent external cohort.

## Materials and methods

### Study design and patient selection

This multicenter retrospective study enrolled 274 patients diagnosed with small bowel obstruction (SBO) who received treatment at three hospitals between January 2015 and December 2023. In the total cohort, 162 patients presented with adhesive obstruction, 69 with internal hernia, and 43 with inguinal hernia. Of these patients, 148 who underwent surgical intervention were assigned to the high-risk group, whereas the remaining 126 patients who achieved clinical improvement following conservative management constituted the low-risk group. Hospital A contributed 198 patients, who were randomly split 7:3 into a training cohort (*n* = 138) and a test cohort (*n* = 60); the remaining 76 patients from Hospitals B and C formed the external validation cohort. Patients were classified as high risk if they underwent surgery or required surgery after failed conservative management and as low risk if they responded well to conservative treatment. Surgical indications were evaluated from the medical and operative records using prespecified criteria, including peritoneal signs, CT evidence of strangulation, ischemia or closed-loop obstruction, hemodynamic deterioration, and failure of nonoperative management. Among the high-risk patients, underwent immediate surgery and underwent surgery after failed nonoperative management (Figure 1).

**Figure 1:**
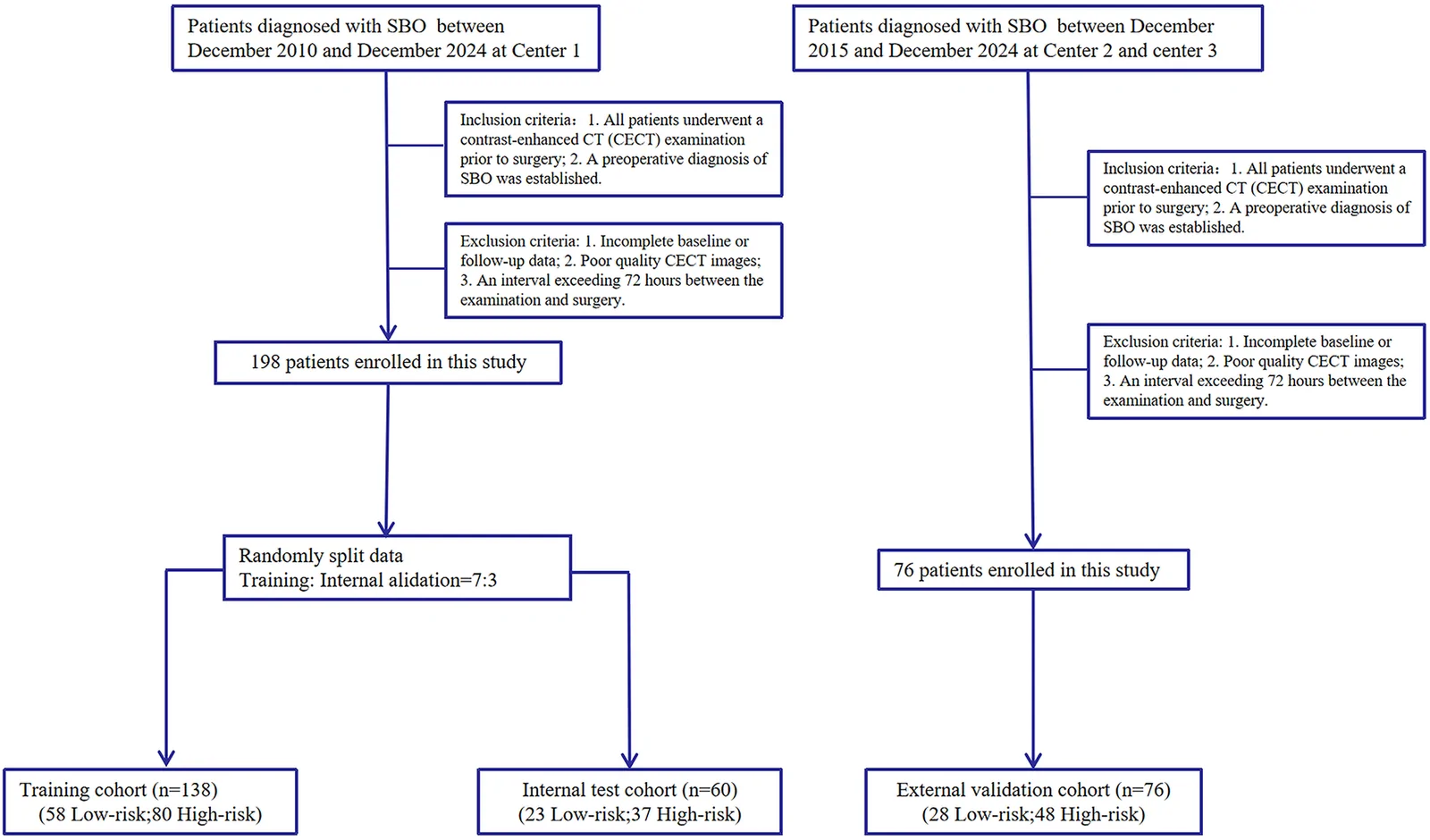
Flowchart illustrating study enrollment for patients with small bowel obstruction (SBO) at three centers, detailing inclusion and exclusion criteria, enrollment numbers, random data split, and division into training, internal test, and external validation cohorts with low-risk and high-risk groups.

The inclusion criteria were as follows: patients with preoperative small bowel obstruction (SBO), preoperative contrast-enhanced computed tomography (CT) examinations, and complete clinical and imaging datasets. Poor image quality was defined as severe motion artifacts, inadequate venous phase enhancement, or incomplete coverage of the lesional intestinal segments, which precluded reliable CT evaluation or radiomic segmentation. Image quality was independently assessed by two abdominal radiologists, and any disagreements were resolved via consensus. Patients were also excluded if the time interval between CT scanning and the definitive treatment decision exceeded 72 h.

The institutional review boards of the participating hospitals approved the study protocol and waived the requirement for informed consent because of the retrospective design. The study was prepared with reference to the STROBE and CLAIM recommendations for observational and machine-learning research.

### Clinical and CT signs

Demographic, clinical, vital-sign, and laboratory variables were collected, including sex, age, white blood cell count, C-reactive protein, blood pressure, heart rate, respiratory rate, abdominal pain pattern, and cessation of flatus or defecation. CT variables included bowel wall thickening, decreased enhancement, maximum bowel diameter, the small-bowel feces sign, mesenteric edema, mesenteric fluid, and ascites. These candidate variables were selected to capture clinically relevant hemodynamic status, inflammatory burden, and CT signs of bowel compromise.

### CT signs, ROI segmentation, and feature extraction

All images were reviewed by two abdominal radiologists with 15 and 10 years of experience, respectively, who were blinded to outcome. Disagreements were resolved by consensus, and uncertain obstruction sites were adjudicated by a senior abdominal radiologist with 30 years of experience.

All patients underwent contrast-enhanced CT, and venous-phase source images in Digital Imaging and Communications in Medicine format were used for radiomics analysis. Regions or volumes of interest were manually delineated around the bowel segment related to obstruction to encompass the affected bowel wall and key pathologic regions while excluding obvious luminal contents, gas, and ascites whenever possible.

After standardized image preprocessing, radiomics features were extracted, including first-order, texture, shape, wavelet, and other filter-derived features. Feature robustness was assessed before model development so that reproducible features could be retained.

### Radiomics reproducibility details

Radiomics feature extraction was performed using PyRadiomics v3.0.1, following the IBSI-1 standard configuration. Six feature classes were computed: first-order statistics, shape-based features, gray-level co-occurrence matrix (GLCM), gray-level run-length matrix (GLRLM), gray-level size-zone matrix (GLSZM), gray-level dependence matrix (GLDM), and neighborhood gray-tone difference matrix (NGTDM). Images were resampled to 1 × 1 × 1 mm^3^ voxel size (B-spline for image, nearest-neighbour for segmentation mask), with intensity discretization using a bin width of 25 HU. No additional filtering was performed. Feature robustness was assessed by ICC using repeated manual segmentations; only features with ICC ≥0.75 were kept. For machine-learning preparation, Z-score normalization was performed using only training-set statistics to avoid leakage. Features with >5% missing voxels were excluded and minor missing entries were median-imputed. Class imbalance was addressed via balanced class weights within the classifier. Hyperparameters were optimized via stratified 5-fold cross-validation on the training cohort (Figure 2).

**Figure 2:**
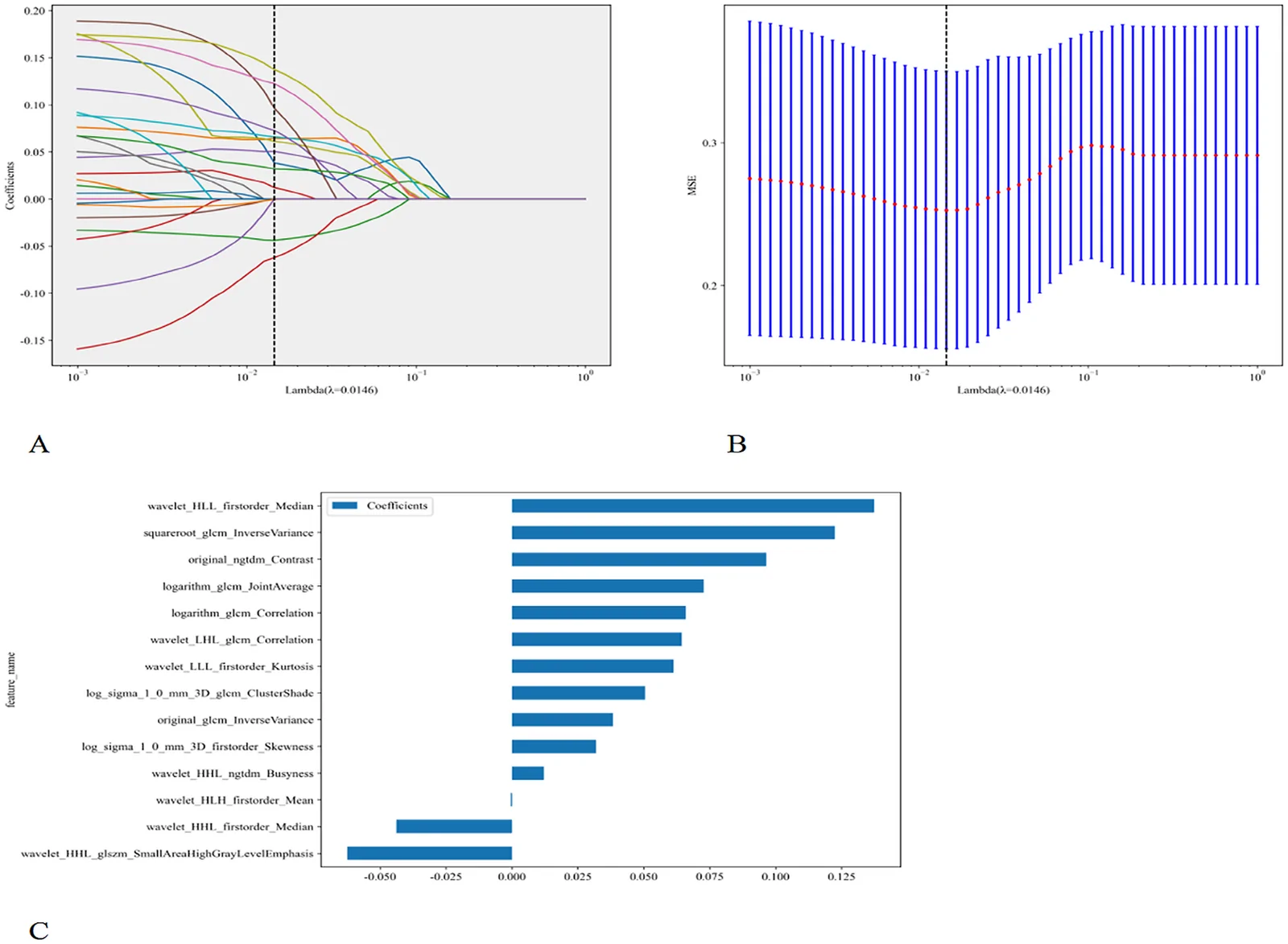
Panel A shows a line plot with multiple colored coefficient paths shrinking towards zero as the penalty parameter lambda increases, with a vertical dashed line marking lambda selection. Panel B contains a mean squared error plot versus lambda, with error bars and a dashed line at the selected lambda. Panel C displays a horizontal bar chart ranking feature names by coefficient values, with the highest absolute values at the top and bottom.

### Model development and interpretability analysis

Radiomics features in the training cohort were standardized before further analysis. For each pair of redundant features with an absolute Pearson correlation coefficient greater than 0.9, one feature was retained. Least absolute shrinkage and selection operator regression with cross-validation was then used to identify features with nonzero coefficients; 14 features were retained. Based on these features, five machine-learning classifiers were developed: logistic regression, naive Bayes, Extra Trees, Light Gradient Boosting Machine (LightGBM), and multilayer perceptron.

Univariable and multivariable logistic regression analyses were used to identify independent clinical predictors and construct the clinical model. These predictors were then integrated with radiomics features to develop the combined model. The best-performing radiomics classifier was further interpreted using SHapley Additive exPlanations (SHAP) summary and feature-importance analyses together with representative case-level visualizations.

### Statistical analysis

Continuous variables were expressed as mean and standard deviation, and categorical variables were expressed as counts and percentages. Group comparisons were performed using the chi-square test, Fisher exact test, or appropriate parametric or nonparametric tests. Model performance was evaluated using AUC, accuracy, sensitivity, specificity, positive predictive value, and negative predictive value. A two-sided *p* value < 0.05 was considered statistically significant.

## Results

### Patient characteristics

A total of 274 patients were included across the training, test, and external validation cohorts. Baseline characteristics were generally similar between the training and test cohorts, with significant differences only in cessation of defecation (*p* = 0.035) and the feces sign (*p* = 0.038). Compared with the training cohort, the external validation cohort differed in respiratory rate (*p* < 0.001), cause of obstruction (*p* = 0.017), colicky pain (*p* = 0.023), continuous pain (*p* < 0.001), mild tenderness (*p* = 0.001), rebound tenderness (*p* < 0.001), decreased enhancement (*p* = 0.049), and mesenteric edema (*p* = 0.006), indicating moderate intercenter heterogeneity.

#### Performance of radiomics models

In the training cohort, LightGBM showed the best overall performance among the radiomics models, with an accuracy of 0.877, an AUC of 0.934, a sensitivity of 0.937, a specificity of 0.793, a positive predictive value of 0.862, and a negative predictive value of 0.902. Extra Trees ranked second with an AUC of 0.917, whereas logistic regression, multilayer perceptron, and naive Bayes yielded AUCs of 0.890, 0.877, and 0.811, respectively.

In the test cohort, LightGBM again achieved the highest AUC (0.870), indicating good internal generalizability, whereas naive Bayes achieved the highest accuracy (0.817). The AUCs of Extra Trees, logistic regression, and multilayer perceptron were 0.814, 0.793, and 0.781, respectively.

In the external validation cohort, naive Bayes showed the best performance, with an AUC of 0.825 and an accuracy of 0.803, whereas LightGBM achieved an AUC of 0.816 and an accuracy of 0.789. Overall, LightGBM was the most stable model across the development cohorts, whereas naive Bayes performed slightly better in external validation.

### Independent clinical predictors and model comparison

Univariable analysis identified age, systolic blood pressure, diastolic blood pressure, white blood cell count, cause of obstruction, vomiting, nausea, peritoneal fluid, colicky pain, continuous abdominal pain, bowel diameter, bowel wall thickening, mesenteric fluid, the feces sign, cessation of defecation, cessation of flatus, and decreased enhancement as variables associated with high-risk status.

In multivariable logistic regression, only diastolic blood pressure and bowel wall thickening remained significant. Lower diastolic blood pressure was associated with high-risk status (OR, 0.955; 95% CI, 0.924-0.988; *p* = 0.024), whereas bowel wall thickening was an independent positive predictor (OR, 1.486; 95% CI, 1.188-1.861; *p* = 0.004).

In the training cohort, the AUCs of the clinical, radiomics, and combined models were 0.848, 0.934, and 0.942, respectively. The combined model performed best, with an accuracy of 0.877, a sensitivity of 0.887, and a specificity of 0.862.

In the test cohort, the AUCs of the clinical, radiomics, and combined models were 0.739, 0.870, and 0.867, respectively. The combined model achieved the highest accuracy (0.817), whereas the radiomics model had the highest AUC.

In the external validation cohort, the AUCs of the clinical, radiomics, and combined models were 0.731, 0.803, and 0.803, respectively. The combined model yielded the highest accuracy (0.833) and sensitivity (0.946), suggesting greater safety for screening high-risk patients (Figure 3).

**Figure 3:**
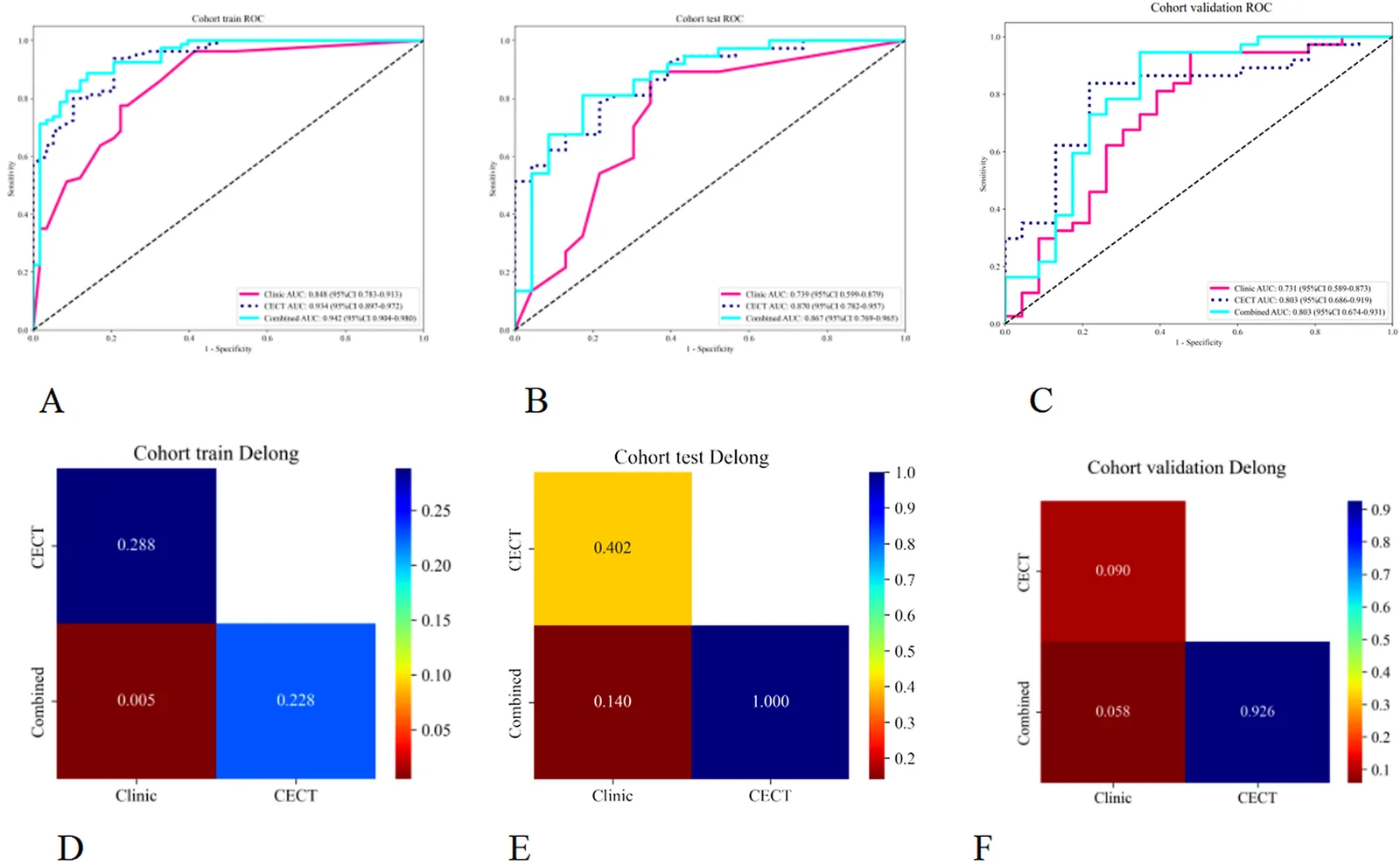
Panel A shows a line chart ROC curve for cohort train with AUC values for Clinic, Radiomics, and Combined models. Panel B shows a ROC curve for cohort test. Panel C shows a ROC curve for cohort validation. Panels D, E, and F display color-coded Delong test heatmaps for cohort train, test, and validation, with values comparing Clinic, Radiomics, and Combined models.

Calibration plots showed that the predicted probabilities of the clinical, radiomics, and combined models generally followed the observed event rates in the training, internal test, and external validation cohorts (Appendix Figure 6, panels D-F), although some deviation was apparent in individual probability bins, particularly in the smaller validation cohorts. These findings indicate that the models provide potentially informative risk estimates, but the probabilities should be interpreted as decision-support values rather than fixed treatment thresholds until prospective recalibration is performed (Figure 4).

**Figure 4:**
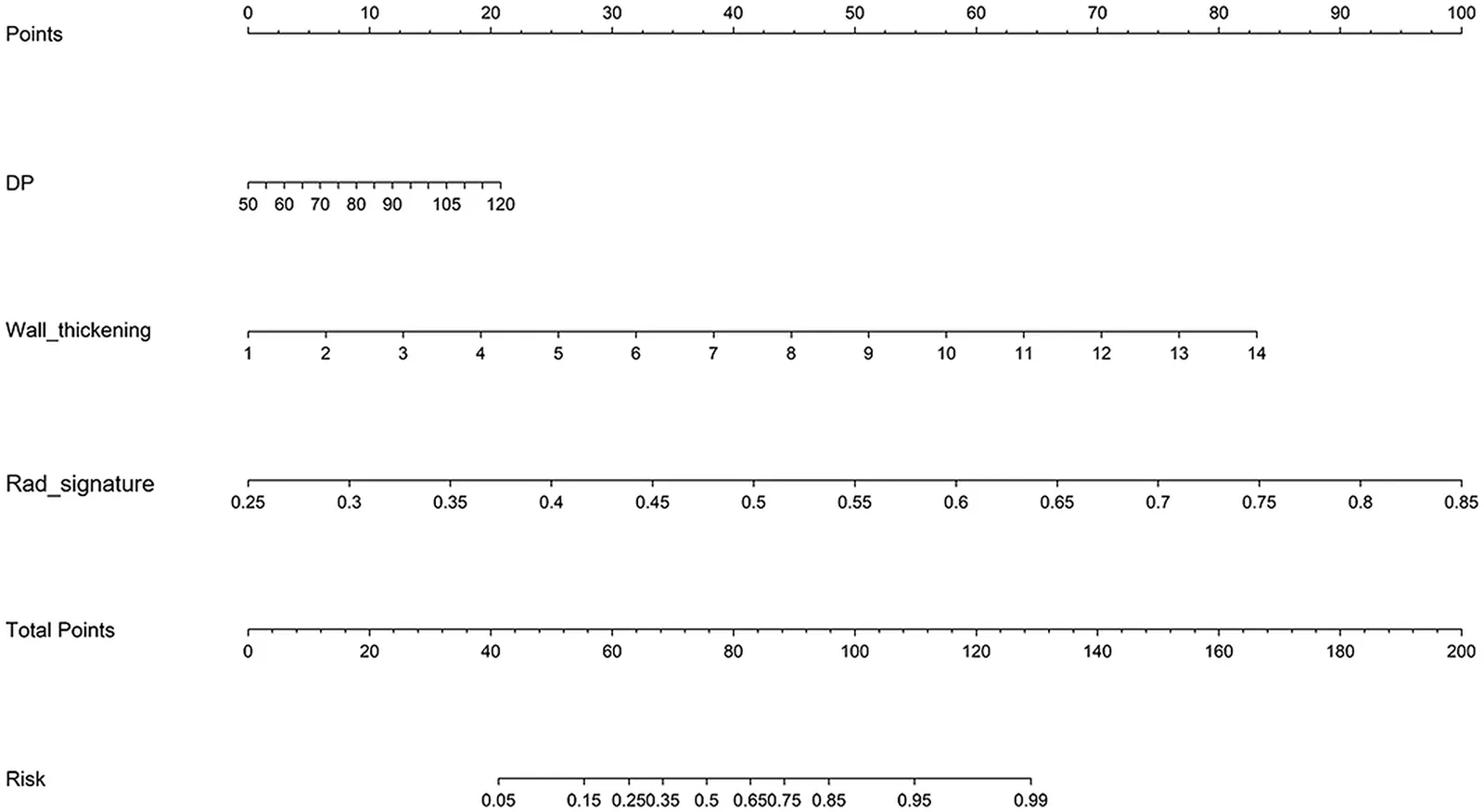
Nomogram graphic presenting six horizontal axes labeled Points, DP, Wall_thickening, Rad_signature, Total Points, and Risk, each with corresponding numerical scales arrayed for medical or statistical risk estimation purposes.

### Model interpretability

SHAP analysis identified wavelet_LHL_glcm_Correlation, wavelet_HLL_firstorder_Median, and logarithm_glcm_Correlation as the three most influential radiomics features. Global distribution plots showed clear differences in key texture features between the high-risk and low-risk groups. Representative-case analyses further showed that the model correctly classified both high-risk and low-risk patients while providing feature-level explanations for each prediction (Figure 5).

**Figure 5:**
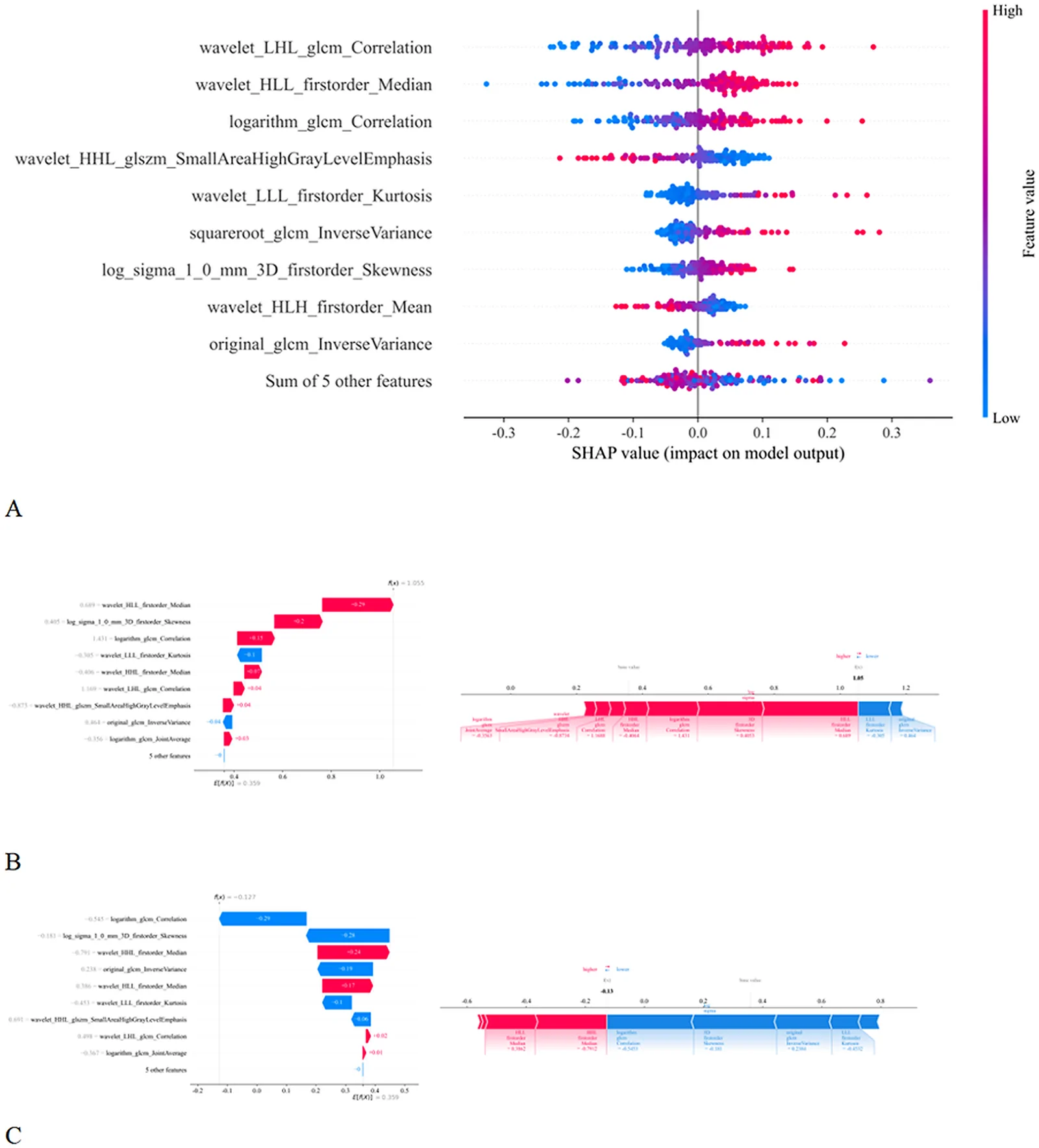
Panel A displays a SHAP summary plot of feature importance with individual feature values shown in blue to red, while panels B and C show SHAP force and bar plots for individual predictions, illustrating positive and negative feature impacts on model output.

**Figure 6:**
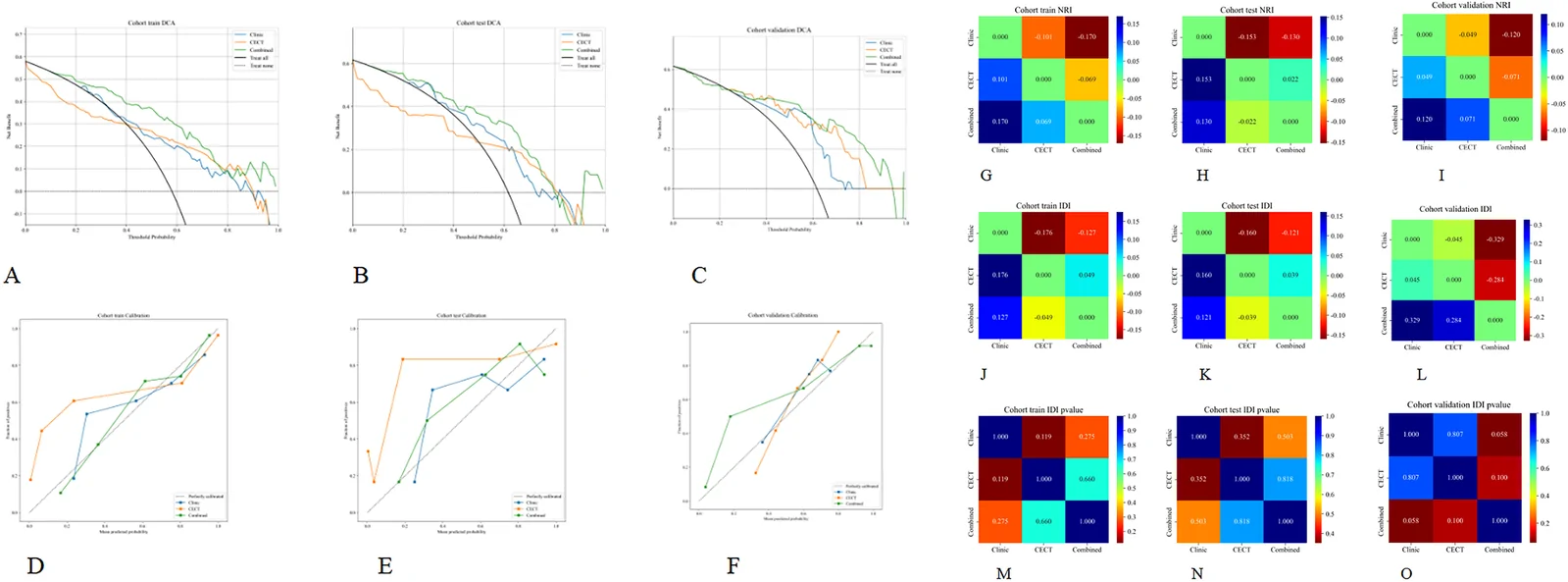
Panel of fifteen images labeled A to O, including line graphs, calibration plots, and heatmaps comparing clinical, CECT, and combined models across train, test, and validation cohorts. Panels A to C show line graphs of decision curve analysis with net benefit against threshold probability. Panels D to F display calibration plots of predicted versus observed probabilities. Panels G to I present cohort net reclassification improvement (NRI) heatmaps, while panels J to L show integrated discrimination improvement (IDI) values. Panels M to O illustrate p-values for IDI comparisons. Color scales indicate performance differences between models.

## Discussion

The principal finding of this study is that CT-based radiomics models can effectively distinguish patients with SBO at high risk, whereas the combined model showed the most clinically useful balance of performance in external validation. Specifically, the combined model achieved AUCs of 0.942, 0.867, and 0.803 in the training, test, and external validation cohorts, respectively, and reached an external-validation accuracy of 0.833 with a sensitivity of 0.946. These data suggest that adding key clinical information to radiomics did not simply improve apparent discrimination in the development cohort, but helped preserve detection of high-risk patients when the model was applied to more heterogeneous cases. Among the candidate algorithms, LightGBM was the most stable radiomics classifier in the training and test cohorts, with AUCs of 0.934 and 0.870, indicating strong adaptability to high-dimensional radiomics features and nonlinear relationships. Importantly, the present study did not define high-risk status only by a single imaging sign or a narrow pathologic endpoint; instead, it reflected a clinically meaningful group of patients who underwent surgery or ultimately required surgical intervention after failed conservative management. From a translational perspective, this framing is relevant because the central question in daily care is often not simply whether one CT sign of ischemia is present, but whether the overall patient is likely to benefit from escalation of management.

These findings are broadly consistent with prior CT-based studies. Previous reports have shown that bowel wall thickening, decreased enhancement, mesenteric abnormalities, and ascites are closely associated with strangulation, ischemia, and failure of conservative management (9–14). Those studies established the current framework for clinical imaging assessment, but they still rely largely on subjective recognition of visible findings and therefore remain limited by interobserver variability and incomplete characterization of subtle intralesional heterogeneity. Our results suggest that radiomics may provide incremental information beyond conventional CT signs, particularly for patients whose routine imaging findings are not yet overtly alarming despite genuinely high risk. One plausible explanation is that radiomics captures spatial complexity, grayscale variation, and textural relationships that may reflect microstructural disorganization, edema, perfusion disturbance, or altered bowel wall composition before those processes become obvious on routine visual inspection. In this sense, radiomics should not be viewed as replacing conventional abdominal CT interpretation, but as extending it with reproducible quantitative descriptors that can enrich clinical judgment in equivocal cases.

Earlier prediction tools relied mainly on clinical variables and visually assessed CT signs. In 233 consecutive patients, Schwenter et al. (23) developed a six-item clinicoradiological score for ischemia requiring bowel resection; a score of at least 3 yielded an AUC of 0.87, sensitivity of 67.7%, and specificity of 90.8%. Although clinically interpretable, that score was derived from conventional variables in a single cohort and had only moderate sensitivity. Paisant et al. (14) subsequently evaluated the Angers CT score prospectively across 15 centers and found that it was associated with failure of conservative management. However, the score remained dependent on predefined visual CT signs and was designed specifically for adhesive SBO. The present study extends these efforts by quantifying intralesional image heterogeneity, comparing multiple classifiers, and testing the models in an external cohort from separate institutions.

Machine-learning studies have addressed related but narrower endpoints. Goyal et al. (21) combined CT and clinical findings in 223 surgically evaluated patients with suspected closed-loop SBO; their internally split random-forest model achieved an AUC of 0.73, but the surgical case spectrum and absence of independent external validation limited generalizability. The prospective multicenter STRISK and NOFA scores focused on immediate surgery and failure of nonoperative treatment in adhesive SBO (26), improving clinical triage but not assessing high-dimensional CT texture. Bunino et al. (27) reported differences in entropy, uniformity, and energy between conservative and operative groups, providing preliminary evidence for SBO radiomics; however, that single-center study included only 55 patients and did not externally validate a multialgorithm model. In contrast, the present study includes multiple SBO causes, directly compares five algorithms, adds SHAP-based global and patient-level explanations, and evaluates performance in an external cohort. The variation in algorithm ranking between internal and external cohorts further shows why independent validation is necessary rather than relying on development-set performance alone.

The study (28) addressed an overlapping question but had important design differences. Wang et al. (29) analyzed 188 patients with adhesive SBO from a single hospital, all of whom underwent surgery, and used an internally split training/test design to predict bowel resection with a radiomic nomogram. The present study addresses a broader triage endpoint—surgery or failure of conservative management versus successful conservative treatment—across adhesive obstruction, internal hernia, and inguinal hernia; it compares five classifiers, evaluates clinical, radiomics, and combined models in parallel, and tests generalizability in an independent cohort from two additional hospitals. SHAP also provides global and case-level explanations.

The multicenter structure of our study also deserves emphasis because the external cohort was not merely a random hold-out subset, but a clinically different population from separate hospitals. Compared with the training cohort, the external validation cohort showed significant differences in respiratory rate, cause of obstruction, colicky pain, continuous pain, mild tenderness, rebound tenderness, decreased enhancement, and mesenteric edema, indicating meaningful intercenter heterogeneity. Against this background, some decrease in discrimination was expected when moving from development to external testing. Even so, the combined model maintained an AUC of 0.803 and achieved the highest external accuracy (0.833) and sensitivity (0.946), while the radiomics-only model had the same external AUC but lower accuracy. These findings suggest that the model captured patterns with practical generalizability rather than only center-specific noise. In other words, the value of the present study lies not in reporting an unusually high single-center AUC, but in showing that clinically useful risk stratification can be retained under more heterogeneous multicenter conditions.

Multivariable analysis provided additional clinically relevant insights that also help explain why the combined model performed better than the clinical model alone. Bowel wall thickening remained an independent predictor with an odds ratio of 1.486 (95% CI, 1.188-1.861; *p* = 0.004), which has clear radiologic-pathologic plausibility because it may reflect bowel wall edema, impaired venous return, and progressive ischemic injury. In contrast, lower diastolic blood pressure was independently associated with high-risk status, with an odds ratio of 0.955 (95% CI, 0.924-0.988; *p* = 0.024), suggesting that the transition to a high-risk state is influenced not only by local morphologic change but also by systemic perfusion status. This interpretation is supported by the model comparison results: in the external validation cohort, the clinical model achieved an AUC of 0.731, whereas the combined model reached an AUC of 0.803 with markedly higher sensitivity. Thus, the data from our study indicate that bedside physiologic information and image-derived features should be interpreted together rather than as competing sources of evidence.

Notably, the combined model did not yield a clearly higher AUC than the radiomics model in external validation, but it improved the metrics that may matter most in emergency decision-making. In the external cohort, the combined and radiomics models both yielded an AUC of 0.803, yet the combined model achieved higher accuracy (0.833) and a sensitivity of 0.946, indicating fewer missed high-risk cases. From a clinical standpoint, this distinction is important. For triage problems such as SBO, a model that misses fewer patients who will ultimately require surgery may be more useful than one that yields only a slightly higher summary discrimination metric. Our data therefore suggest that the combined model may be better suited for identifying patients who need closer observation, earlier surgical consultation, or a lower threshold for intervention, even when its AUC is not dramatically different from that of the radiomics-only approach.

Interpretability remains a central issue in the clinical translation of radiomics and machine learning (19, 20). Through SHAP analysis, we identified wavelet_LHL_glcm_Correlation, wavelet_HLL_firstorder_Median, and logarithm_glcm_Correlation as the three most influential radiomics features and visualized their contributions at both the population and individual levels. These features describe local textural correlation, grayscale-distribution characteristics, and heterogeneity and may be related to bowel wall disorganization, perfusion disturbance, or increased tissue complexity in compromised segments. However, this biologic interpretation is hypothesis-generating because no direct histopathologic or perfusion reference standard was available. Future studies correlating these radiomics features with resection histopathology, perfusion markers, and standardized CT signs are needed to confirm their clinical meaning. Accordingly, SHAP improves transparency regarding how the model reached a prediction, but it does not by itself establish the underlying tissue mechanism.

At the point of care, feature-level SHAP explanations could allow radiologists and surgeons to determine whether a high-risk prediction is driven by clinically plausible image patterns, compare the model output with conventional CT findings, and identify discordant cases that warrant additional review. This traceability may increase confidence in using the model as decision support while preserving clinician oversight rather than treating the algorithm as an autonomous recommendation.

Another point worth emphasizing is that external validation in our study also revealed meaningful differences in algorithm behavior. LightGBM was the most stable radiomics model in the development cohorts, but naive Bayes showed the best external AUC at 0.825 with an accuracy of 0.803. This shift indicates that algorithm ranking was sensitive to changes in case mix and data distribution across centers. Rather than viewing this as a weakness, we consider it a realistic finding for medical artificial intelligence research. It suggests that no single classifier should be assumed to be universally optimal on the basis of internal performance alone and highlights the importance of comparing multiple algorithms within the same dataset. Future studies should therefore focus not only on improving discrimination, but also on calibration, threshold selection, workflow integration, and prospective evaluation of whether model-assisted care can reduce delays in recognizing patients who need urgent intervention.

Clinical implementation will require attention to workflow speed. Manual segmentation by experienced radiologists is feasible for retrospective model development but may be impractical in time-sensitive emergency settings because it adds operator time and may introduce interobserver variation. A pragmatic deployment pathway would use the model as a decision-support layer after rapid CT interpretation, with semi-automated or automated bowel-segment localization and contouring followed by radiologist review of the proposed region of interest. Prospective studies should quantify segmentation time, correction burden, failure rates, and model performance across centers before routine use.

Although the independent external cohort tested transportability across institutions with different case mix, formal subgroup stability by sex, age, and etiology remains to be quantified from patient-level predictions. Estimates in smaller etiologic strata, particularly the inguinal hernia subgroup, may be imprecise and should be interpreted as exploratory.

This study has several limitations. First, the retrospective design introduces selection bias; in particular, excluding examinations with severe artifacts, inadequate enhancement, or incomplete bowel coverage may underrepresent technically challenging real-world cases. Second, the sample size remained limited, and heterogeneity between the training and external validation cohorts may have affected model stability. Third, the workflow relied mainly on traditional machine-learning methods and manual lesion delineation rather than deep learning, automated segmentation, or longitudinal clinical variables. Fourth, the outcome was defined by the need for surgery or failure of conservative management. Although operative decisions were reviewed against prespecified clinical and imaging indications, surgeon preference, institutional protocols, bed availability, comorbidity, and patient preference may have varied across centers and introduced practice-pattern variation into outcome labeling. Fifth, the proposed biologic interpretation of the leading radiomics features was not validated against histopathology. Larger prospective multicenter studies using standardized surgical indications, explicit timing measures, prespecified image-quality criteria, and histopathologic correlation are needed to validate the model and quantify its clinical decision impact.

## Conclusion

In conclusion, CT-based radiomics showed good ability to identify patients with SBO at high risk. A combined model incorporating radiomics and key clinical predictors improved external validation accuracy and sensitivity and may help guide early triage and intervention. If further validated prospectively, this type of interpretable imaging biomarker could support more standardized communication between radiologists and surgeons, improve prioritization of patients who warrant closer observation, and contribute to safer decision-making in a time-sensitive emergency setting.

## Data Availability

The data analyzed in this study is subject to the following licenses/restrictions: The data are not publicly available due to patient privacy concerns. Requests for access should be directed to the corresponding authors. Requests to access these datasets should be directed to sara_good@126.com.
